# Transfusional Approach in Multi-Ethnic Sickle Cell Patients: Real-World Practice Data From a Multicenter Survey in Italy

**DOI:** 10.3389/fmed.2022.832154

**Published:** 2022-03-16

**Authors:** Giovanna Graziadei, Lucia De Franceschi, Laura Sainati, Donatella Venturelli, Nicoletta Masera, Piero Bonomo, Aurora Vassanelli, Maddalena Casale, Gianluca Lodi, Vincenzo Voi, Paolo Rigano, Valeria Maria Pinto, Alessandra Quota, Lucia D. Notarangelo, Giovanna Russo, Massimo Allò, Rosamaria Rosso, Domenico D'Ascola, Elena Facchini, Silvia Macchi, Francesco Arcioni, Federico Bonetti, Enza Rossi, Antonella Sau, Saveria Campisi, Gloria Colarusso, Fiorina Giona, Roberto Lisi, Paola Giordano, Gianluca Boscarol, Aldo Filosa, Sarah Marktel, Paola Maroni, Mauro Murgia, Raffaella Origa, Filomena Longo, Marta Bortolotti, Raffaella Colombatti, Rosario Di Maggio, Raffaella Mariani, Alberto Piperno, Paola Corti, Carmelo Fidone, Giovanni Palazzi, Luca Badalamenti, Barbara Gianesin, Frédéric B. Piel, Gian Luca Forni

**Affiliations:** ^1^Rare Diseases Center, General Medicine Unit, Fondazione IRCCS Ca' Granda Ospedale Maggiore Policlinico, Milan, Italy; ^2^Department of Medicine, University of Verona and AOUI Verona, Verona, Italy; ^3^Dipartimento della Salute della Donna e del Bambino Azienda Ospedaliera, Azienda Ospedaliera Universitaria, Padova, Italy; ^4^Servizio Immunotrasfusionale, Azienda Ospedaliero Universitaria Modena, Modena, Italy; ^5^Pediatric Clinic Hemato-Oncology Department, University of Milano-Bicocca, MBBM Foundation, San Gerardo Hospital, Monza, Italy; ^6^Servizio Immunotrasfusionale, Azienda Ospedaliera Maria Paternò Arezzo, Ragusa, Italy; ^7^UOC Medicina Trasfusionale, Azienda Ospedaliera Universitaria, Verona, Italy; ^8^Department of Women, Child and General and Specialized Surgery, University “Luigi Vanvitelli”, Naples, Italy; ^9^Medicina Trasfusionale, Azienda Ospedaliera Universitaria Sant'Anna, Ferrara, Italy; ^10^Centro per le Emoglobinopatie – Dipartimento di Scienze Cliniche e Biologiche, Università di Torino, Ospedale San Luigi Gonzaga, Torino, Italy; ^11^Campus of Haematology Franco e Piera Cutino, A.O.O.R. “Villa Sofia-Cervello” di Palermo, Palermo, Italy; ^12^Hematology, Thalassemia and Congenital Anemia Center, Ospedale Galliera, Genova, Italy; ^13^Unità Operativa Semplice Dipartimentale Talassemia P.O. Vittorio Emanuele, Gela, Italy; ^14^Italian Association of Pediatric Hematology Oncology (AIEOP) Coagulation Disorders Working Group, Brescia, Italy; ^15^Pediatric Hematology/Oncology Unit, Università di Catania, Catania, Italy; ^16^Servizio Microcitemia, Presidio Ospedaliero SL 5, Crotone, Italy; ^17^UOSD di Talassemia ed Emoglobinopatie, Azienda Ospedaliero-Universitaria Policlinico San Marco, Catania, Italy; ^18^Centro Microcitemie, Azienda Ospedaliera “Bianchi-Melacrino-Morelli”, Reggio Calabria, Italy; ^19^SSD Oncoematologia Pediatrica - Policlinico di S.Orsola, Bologna, Italy; ^20^Servizio Trasfusionale, Ospedale Santa Maria delle Croci, Ravenna, Italy; ^21^Ospedale Santa Maria della Misericordia, Perugia, Italy; ^22^Policlinico San Matteo di Pavia, Pavia, Italy; ^23^Unità Operativa Ematologia, Centro di Microcitemia, Azienda Ospedaliera di Cosenza, Presidio Ospedaliero “Annunziata” Cosenza, Cosenza, Italy; ^24^Ospedale Spirito Santo, Pescara, Italy; ^25^Department of Talassemia, Siracusa Hospital, Siracusa, Italy; ^26^SOC Pediatria Nuovo Ospedale Prato Santo Stefano, Prato, Italy; ^27^Hematology, Department of Translational and Precision Medicine, Sapienza University, Rome, Italy; ^28^Unità Operativa Dipartimentale Talassemia, Presidio Garibaldi-Centro ARNAS Garibaldi, Catania, Italy; ^29^UOC Pediatria Universitaria, Università di Bari, Bari, Italy; ^30^Ospedale di Bolzano, Bolzan, Italy; ^31^UOSD Malattie rare del globulo rosso, AORN A. Cardarelli, Naples, Italy; ^32^Hematology and Bone Marrow Transplant Unit, IRCCS San Raffaele Scientific Institute, Milan, Italy; ^33^Servizio di Immunoematologia e Medicina Trasfusionale, ASST Sette Laghi, Varese, Italy; ^34^Centro Provinciale per le Microcitemia, Ospedale San Martino di Oristano, Oristano, Italy; ^35^Ospedale Pediatrico Microcitemico, Università di Cagliari, Cagliari, Italy; ^36^Department of Oncology and Oncohematology, University of Milan, Milan, Italy; ^37^Rare Disease Centre - Hereditary anemias - ASST-Monza, S. Gerardo Hospital - University of Milano-Bicocca, Monza, Italy; ^38^Biomedicina, Neuroscienze e Diagnostica avanzata, University of Palermo, Palermo, Italy; ^39^ForAnemia Foundation, Genoa, Italy; ^40^Department of Epidemiology and Biostatistics, Imperial College London, London, United Kingdom

**Keywords:** hydroxycarbamide, multi-ethnicity, sickle cell disease, transfusion therapy, alloimmunization

## Abstract

Sickle cell disease (SCD) is a worldwide distributed hereditary red cell disorder characterized by recurrent acute vaso-occlusive crises (VOCs and anemia). Gold standard treatments are hydroxycarbamide (HC) and/or different red blood cell (RBC) transfusion regimens to limit disease progression. Here, we report a retrospective study on 1,579 SCD patients (median age 23 years; 802 males/777 females), referring to 34 comprehensive Italian centers for hemoglobinopathies. Although we observed a similar proportion of Caucasian (47.9%) and African (48.7%) patients, Italian SCD patients clustered into two distinct overall groups: children of African descent and adults of Caucasian descent. We found a subset of SCD patients requiring more intensive therapy with a combination of HC plus chronic transfusion regimen, due to partial failure of HC treatment alone in preventing or reducing sickle cell-related acute manifestations. Notably, we observed a higher use of acute transfusion approaches for SCD patients of African descent when compared to Caucasian subjects. This might be related to (i) age of starting HC treatment; (ii) patients' low social status; (iii) patients' limited access to family practitioners; or (iv) discrimination. In our cohort, alloimmunization was documented in 135 patients (8.5%) and was more common in Caucasians (10.3%) than in Africans (6.6%). Alloimmunization was similar in male and female and more frequent in adults than in children. Our study reinforces the importance of donor-recipient exact matching for ABO, Rhesus, and Kell antigen systems for RBC compatibility as a winning strategy to avoid or limit alloimmunization events that negatively impact the clinical management of SCD-related severe complications.

**Clinical Trial Registration:**

ClinicalTrials.gov, identifier: NCT03397017.

## Introduction

Sickle cell disease (SCD) is one of the most common inherited red cell disorders worldwide, recognized by the World Health Organization (WHO) and the United Nations as a “global health problem” (1, 2). About 300,000 infants are born each year with SCD; ~90,000 in Nigeria, 44,000 in India, 40,000 in the Democratic Republic of Congo, 12,000 in the United States (US), 2,000 in Europe, and 300 in the United Kingdom (UK) (3). Although SCD is part of the rare disease group, more than 10,000 SCD patients live in the UK and more than 25,000 in France, making SCD much more common than cystic fibrosis or hemophilia A (4, 5). The median life expectancy is now more than 60 years in Europe (6), but about 80% of patients with SCD in African regions die during childhood (7).

Treatment options for SCD are still limited, particularly in low-income settings. Hydroxycarbamide (HC) is the gold standard treatment for both children and adults with SCD (8). Hydroxycarbamide reduces painful vaso-occlusive events (VOCs) and acute chest syndrome as well as the need for red blood cell (RBC) transfusion. Until now, RBC transfusion has largely been used for clinical management of both acute and chronic sickle cell-related complications (9, 10). Despite the known indications for RBC transfusion and HC therapy (9, 11–16), treatment of patients with SCD in clinical practice is still not homogeneous.

Over the course of a lifetime, patients with SCD might be exposed to different RBC transfusion regimens, ranging from simple transfusion on-demand or manual/automatized RBC exchange to manage acute VOCs or to limit disease progression and organ damage (3, 17). Alloimmunization to RBC blood group antigens carries an increased risk of hemolytic transfusion reactions (DHTR), potentially delaying the identification of compatible RBC units (18, 19). In Europe, Italy presents a distinctive epidemiologic European niche for SCD, characterized by endemic SCD population mainly in Southern Italian regions and multi-ethnic SCD population localized in Central and Northern Italian regions (20–23). This affects the distribution of SCD genotypes, mainly represented by Sβ^0^/Sβ^+^ or SS genotype in patients of Caucasian descent, while SS or SC genotypes characterize patients of African descent. To better understand the scenario of transfusion regimens and alloimmunization of SCD patients in Italy, we carried out a national survey involving the National Comprehensive Reference Centers for SCD and the Italian Society of Thalassemia and Hemoglobinopathies (SITE), in collaboration with the Italian Society of Transfusion Medicine and Immunohematology (SIMTI) and the Italian Association of Hematology and Pediatric Oncology (AIEOP).

## Materials and Methods

### Study Design

This is a retrospective multicenter national study (ClinicalTrials.gov identifier NCT03397017). Data from the clinical records of eligible patients were collected from 2015 to 2018 through a standard web-based application (www.SITE-italia.org) encrypted by the Central Server. The recording of personal, therapy, and complications data of patients with sickle cell anemia included in the National Transfusion Treatment Survey was undertaken by the responsible investigator or sub-investigators selected by each center, following registration on the site. The operator can subsequently access the patient's clinical data and perform updates to follow clinical evolution over time. The study did not involve any additional tests compared to routine patient management.

The study aimed to identify the lifelong therapeutic approaches, focusing on transfusion regimens, in clinical management of a large multi-ethnic Italian cohort of patients with SCD. The study was coordinated by SITE, in collaboration with SIMTI and AIEOP. A total of 34 Italian reference centers from 15 Italian regions were involved. Data were collected through the National Comprehensive Reference Centers for SCD. The study was approved by the Ethics Committee of Fondazione IRCCS Ca' Granda, Ospedale Maggiore Policlinico of Milan, Italy, the coordinating center of the study.

The inclusion and exclusion criteria were, respectively, the presence or absence of a biochemical and/or genetic diagnosis of SCD and the capability or not to give informed consent. Written informed consent was obtained from all included patients after the study aims and protocol had been explained. When necessary, a Cultural Linguistic Mediator could be engaged to assist in the consent process. Besides demographic data, every center collected data concerning the type and sequence of therapy, acute transfusion therapy (ATR), chronic transfusion therapy (CTR, i.e., regular transfusion regimen for more than 1 year), or HC, the main indications for ACR and CTR, and details on alloimmunization (number and systems of antibodies) over the previous 4 years of treatment.

### Statistical Analysis

Descriptive statistics of continuous variables were expressed using medians and interquartile range (IQR: 25th-75th percentile); dichotomous variables were summarized as counts and percentages. The Shapiro-Wilk test was used to test the normality of distribution. As no normal distribution was found, the differences between groups were analyzed with the Wilcoxon signed-rank test. Two- or multiple-proportion comparisons between variables were performed with the *Z*-test. All tests were two-sided, and *p*-values < 0.05 were considered as statistically significant. Data management and graphs were created with Excel (Microsoft, Seattle, WA, USA), and statistical analysis was performed using R version 3.5.1 for Windows (R Core Team, Vienna, Austria).

## Results

### SCD Italian Patients Cluster Into Two Overall Distinct Groups: Children of African Descent and Adults of Caucasian Descent

**Figure 1:**
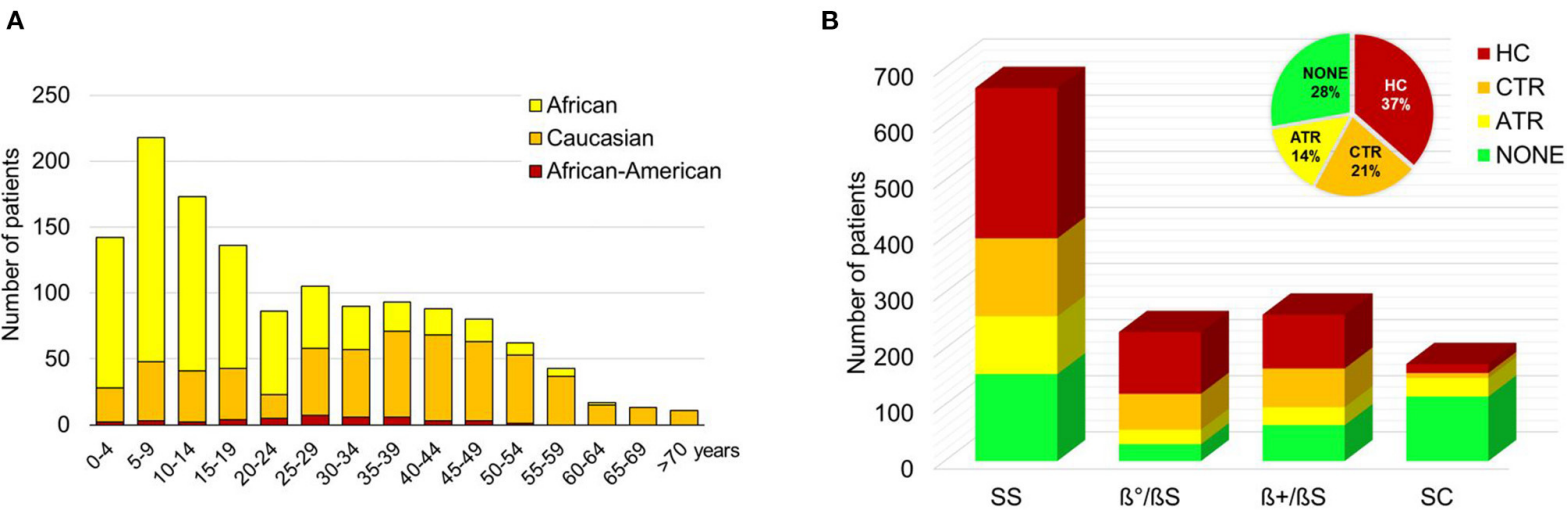
**(A)** Distribution of sickle cell disease (SCD) patients by age and ethnicity. Data are shown as a column chart that reports, for each age group, the number of Africans and Caucasians patients of the cohort. **(B)** Distribution of SCD patients by genotype and therapy. Data are shown as a column chart that reports, for each genotype, the number of patients that received HC/ATR/CTR/NONE as their therapy regimen. ATR, acute transfusion; CTR, chronic transfusion; HC, hydroxycarbamide.

Our study shows that Italian SCD patients overall cluster into two distinct groups: children of African and adults of Caucasian descent (Figure 1A). We collected data from 1,579 patients, of whom 802 were male (51%) and 777 were female (49%) with a median age of 23 (IQR 10–41) years (Supplementary Figure S1). No significant difference in age was found between males and females (*p* = 0.18); the median age was 21.5 (IQR 10–40) years in males and 25.3 (IQR 10–42) years in females. Ethnicity was equally distributed between Caucasians (47.9%) and Africans (48.7%), with a minority of African American (2.8%) and Asian (0.4%) SCD patients. Ethnicity was not defined for 0.2% of patients (Table 1). In African patients, the most common genotype was homozygosity for HbS (SS; 71.3%). Compound heterozygosity with β-thalassemia mutations was 2.3% for S/β0-thalassemia, 3.9% for S/β+-thalassemia, and 20.8% for SC disease. Otherwise, the most prevalent genotype in Caucasians was S/β-thalassemia (74.5%; in particular, S/β^0^-thalassemia 34.8% and S/β^+^-thalassemia 39.7%). SS genotype was present in 21.4% and SC in 0.5% of SCD patients of Caucasian descent. The median age of African SCD patients was 12 (IQR 7–22) years compared with 38 (IQR 25–49) years in Caucasian patients (*p* < 0.001).

**Table 1:** Demographic characteristics of patients with sickle cell disease (SCD).

**Ethnicity**	**Genotype**	**Counts (%)**	**Males (%)**	**Median age (IQR), yr**
African (*n* = 769)	HbSS	548 (71.3)	280 (51.2)	11 (6.9–19.8)
	HbS/β°-thalassemia	18 (2.3)	12 (66.7)	14.5 (9.5–19.8)
	HbS/β^+^-thalassemia	30 (3.9)	16 (53.3)	26.5 (15.6–37.5)
	HbSC	160 (20.8)	78 (48.8)	16.9 (7.8–27.0)
	Not defined	13 (1.7)	3 (23.1)	40 (12–46)
Caucasian (*n* = 756)	HbSS	162 (21.4)	76 (46.9)	35.4 (18–48)
	HbS/β°-thalassemia	263 (34.8)	138 (52.5)	40 (27–50)
	HbS/β^+^-thalassemia	300 (39.7)	161 (53.7)	39 (25–51)
	HbSC	4 (0.5)	3 (75.0)	31.5 (20–40.8)
	Not defined	27 (3.6)	14 (51.9)	31.9 (22–45)
African-American (*n* = 45)	HbSS	24 (53.3)	9 (37.5)	29 (22.6–36.2)
	HbS/β°-thalassemia	4 (8.9)	2 (50.0)	12.5 (6.8–23.2)
	HbS/β^+^-thalassemia	4 (8.9)	1 (25.0)	25 (9.5–41.5)
	HbSC	13 (28.9)	4 (30.8)	27.5 (21–35)

*Asian patients comprised 0.4%, and people of another ethnicity made up the other 0.2%*.

*Hb, hemoglobin; IQR, interquartile range; yr, years*.

### Combined Transfusion Approaches With Hydroxycarbamide Were Used to Limit HC-Resistant Sickle Cell-Related Clinical Manifestations

**Figure 2:**
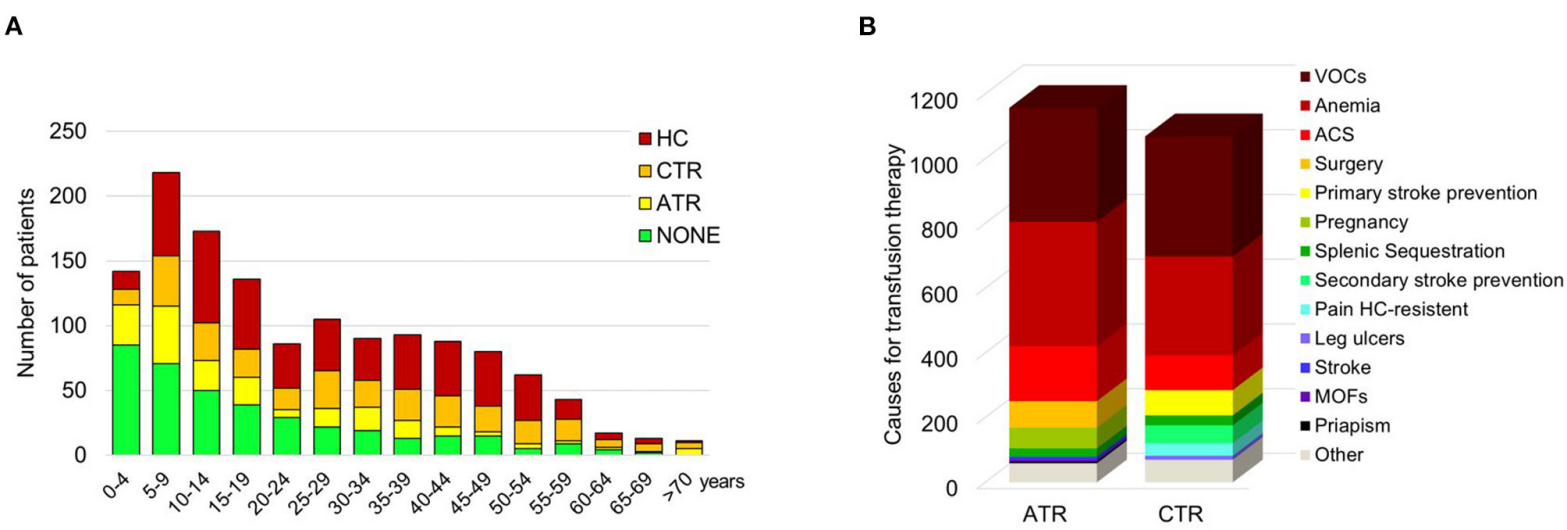
**(A)** Distribution of sickle cell disease (SCD) patients by age and therapy. Data are shown as a column chart that reports, for each age group, the number of patients that received HC/ATR/CTR/NONE as their therapy regimen. **(B)** Distribution of the indications for transfusion therapy. Data are shown as counts of the indications for ATR and CTR. ACS, acute chest syndrome; ATR, acute transfusion; CTR, chronic transfusion; HC, hydroxycarbamide; MOFs, multi-organ failures; VOCs, vaso-occlusive events.

Full data on therapeutic management of SCD patients were collected for 1,364 out of the 1,579 patients (Table 2; Figure 1B; Supplementary Figure S1). The following therapeutic regimens were identified: (i) HC: chronic hydroxycarbamide therapy for more than 1 year; (ii) CTR: chronic transfusion regimen; (iii) ATR: acute transfusion approach; and (iv) no treatment (Figure 2A). When we considered therapeutic strategies in function of age, we found that blood transfusion approaches were used throughout patient's journey (Figure 2B). A total of 292 patients (21.4%) were on CTR, 196 (14.4%) on ATR, and 497 on HC (36.4%), of whom 100 needed transfusions for acute VOCs. Three hundred and seventy-nine (27.8%) patients never received long-term therapy. In both ATR and CTR clinical setting, donor-recipient exact matching for serological ABO, Rhesus and Kell antigen for RBC compatibility was carried out according to international and Italian guidelines, released in 2014 (9, 13, 24–27).

**Table 2:** Treatments of SCD patients (*n* = 1,364) and distribution according to ethnicity and genotype.

**Therapy^a^**	**Pts (%)**	**M vs. F**	**African vs. Caucasian vs. African-American**	**SS, No. (%)**	**S/β°, No. (%)**	**S/β^+^, No. (%)**	**SC, No. (%)**	***p*-value**
CTR	292 (21.4)	152/140	94/181/14	139 (49.5)	64 (22.8)	69 (24.6)	9 (3.2)	<0.001
ATR	196 (14.4)	92/104	116/69/10	103 (53.1)	26 (13.4)	32 (16.5)	33 (17)	<0.001
HC	497 (36.4)	273/224	238/246/11	268 (54.7)	110 (22.4)	96 (19.6)	16 (3.3)	<0.001
None	379 (27.8)	196/183	280/91/7	155 (42.6)	30 (8.2)	64 (17.6)	115 (31.6)	<0.001
^b^ATR/CTR → HC	236 (17.3)	129/107	120/109/7	138	53	37	3	–
^b^HC → CTR	14 (1.0)	8/6	6/7/1	9	2	2	1	–

a *The sum of partial counts may not correspond to the total in the case of different and/or not defined ethnicity/genotype*.

b *Subset of 250 patients for whom it was possible to follow the timing of therapy*.

*A total of 100 patients under HC were transfused for the clinical management of acute vaso-occlusive events (VOCs)*.

*ATR, acute transfusion regimen; CTR, chronic transfusion regimen; F, female; HC, Hydroxycarbamide; M, male; Pts, patients*.

The main indications for CTR were recurrent severe VOCs (371 events; 37.1%), chronic anemia (306 events; 30.6%), primary stroke prevention (78 events, 7.8%) and secondary stroke prevention (55 events, 5.5%), acute chest syndrome (ACS; 107 events; 10.7%), splenic sequestration (31 events, 3.1%), HC-resistant pain (39 events; 3.9%) and leg ulcers (12 events; 1.2%) (Figure 2B). The median value of hemoglobin (Hb) before CTR was 9.2 g/dL (IQR: 8.5–10, min-max: 5.2–14.9 g/dL, data available for 85% of patients) and the median value of HbS was 50% of total Hb (IQR: 39–55, min-max: 8.5–89%, data available for 67% of patients).

The main indications for ATR were acute anemia (384 events; 35%), VOCs (352 events; 32.1%), ACS (170 events; 15.5%), surgery (82 events; 7.5%), pregnancy (64 events; 5.8%), splenic sequestration (26 events; 2.4%), stroke (9 events; 0.8%), multi-organ failure (6 events; 0.5%) and priapism (5 events; 0.5%) (Figure 2B). Before ATR, the median value of Hb level was 8.0 g/dL (IQR: 6.8–9.0 g/dL, min-max 2.7–15.2 g/dL, data available for 72% of patients), with 67% of HbS (IQR: 56–78%, min-max: 30–93, data available for 43% of patients). Patients who were not treated had a median Hb value of 10.7 g/dL (IQR: 9.3–12 g/dL). When we stratified SCD population according to age: children (<18 years) and adults (>18 years), the proportion of different indications for ATR was similar within the two groups except for pregnancy and priapism, which were present in adults (Supplementary Figure S2A). Considering CTR, we found CTR to be more frequently introduced in adults than in children with SCD (Supplementary Figure S2A). CTR protocols were more frequently applied in adults than in children to prevent ACS and to treat chronic pain resistant to HC therapy (Supplementary Figure S2A). We found a higher proportion of Caucasian adults (older than 20 years) under CTR (135/181, 75%) when compared to African adults (41/94, 44%). In children and adolescents (younger than 20 years), the proportion of Caucasians treated with CTR (46/181, 25%) was lower when compared with Africans (53/94, 56%). The ratio between patients in ATR and CTR decreases with during aging (*p* < 0.05, Figure 2A).

HC was generally started after 5 years of age in SCD patients of both Caucasian (6%) and African (94%) descent. Two hundred and fourteen of 246 Caucasian patients were adults (17% SS and 79% Sβ genotypes). As expected, SCD patients with SC genotype showed less intensive medical treatment than SCD patients with either SS or Sβ^0/+^ genotypes (Figure 1B).

For 250 out of 1,364 patients (18.3%), it was possible to follow the temporal sequence of change in therapy (Table 2). Fourteen patients (1.0% of the 1,364) switched from HC to CTR, and 236 patients (17.3%) switched from CTR to HC. A subset of 60 patients (4.3% of the 1,364 with treatment data available) received a simultaneous blood transfusion and HC for a median period of 4.6 years (IQR 2.2–11.1 years). This was due to the severity of sickle cell-related clinical presentation, which was only partially controlled by HC therapy. The main indications for CTR in these 60 patients were, respectively, VOCs (number of events: 33), anemia (*n* = 25), ACS (*n* = 13). HC-resistant pain (*n* = 6), splenic sequestration (*n* = 4), primary (*n* = 2) or secondary (*n* = 3) stroke prevention. Combined therapy (CTR+HC) was started at a median age of 20.5 (IQR 10.7–30.8) years. In 14/250 patients, HC was started at a median age of 30.7 (IQR 28.1–41.4) years, and CTR needed to be added to HC after a mean period of 3.8 (IQR 2.4–5.3) years at a median age of 34.4 (IQR 30.3–48.3) years. The proportion of SCD patients without any treatment decreased with aging (Figure 2A). Two out of 1,364 patients with SCD displayed hyperhemolysis reaction after RBC transfusion (1 African SS child: 5 years of age; 1 African SS adult: 23 years of age).

Collectively, our data indicate that acute and chronic transfusion approaches were chosen to limit disease progression in patients of both Caucasian and African descent. RBCs from Caucasian donors were used. The donor-recipient exact matching for ABO, Rhesus and Kell antigen for RBC compatibility strategy limits alloimmunization, which might further complicate clinical management of patients with SCD (9, 18, 19, 28).

### Low Immunization Events Characterize the Italian Multi-Ethnic Cohort of SCD Patients

In our study, alloimmunization was documented in 135 (8.5%) out of 1,579 patients with SCD. Sixty-one of these 135 patients (45.2%) were already alloimmunized at their arrival at the comprehensive SCD center. Four of them (6.6%) developed alloimmunization against new RBC antigen (Supplementary Figure S3A) 36 of these 135 patients (27%) developed alloantibodies after they arrived at the Italian referral center (Supplementary Figure S3A). Data on alloantibody status in relation to the timing of arrival at the SCD center were not available for the other 38 alloimmunized SCD patients.

Among the alloimmunized SCD patients, 33 (24.4%) were younger than 18 years, 53 (39.3%) were aged 18–40 years, and 49 (36.3%) were older than 40 years (Supplementary Figure S3B). Fifty-one alloimmunized patients were African [median age 20 years (IQR 11–28 years)], corresponding to 6.6% of the African group; 78 patients were Caucasian [median age 41 years (IQR 31–51 years)], representing 10.3% of the Caucasian group; five patients were African American [median age 35 years (IQR 30–37 years)] and for one patient (age 29 years) ethnicity was not reported. Thus, alloimmunization was more frequent in adults than in children with SCD (*p* < 0.001). The frequency of alloimmunization was similar in female and male patients with SCD.

RBC-specific antibodies were detected in 124 patients, of whom 70 (41 males, 29 females) had a single antibody and 43 (16 males and 27 females) had multiple antibodies (59.5%, 28.6, 9.5, and 2.4%, respectively, with two, three, four, and six antibodies). Specific antibodies were not identified in 11 patients (3 males and 8 females) (Supplementary Figure S3C). A higher proportion of Caucasian patients had multiple antibodies compared with patients of other ethnicities. In the Caucasian group, a single antibody was prevalently found in males (24/40, 60%), while there were no significant differences between genders in the African group. When genotypes were considered, we observed alloimmunization to be more frequently associated with SS genotype in both African and African American patients, whereas SS and Sβ^0/+^ genotypes were equally associated with alloimmunization in SCD patients of Caucasian descent (Supplementary Figure S3B).

**Figure 3:**
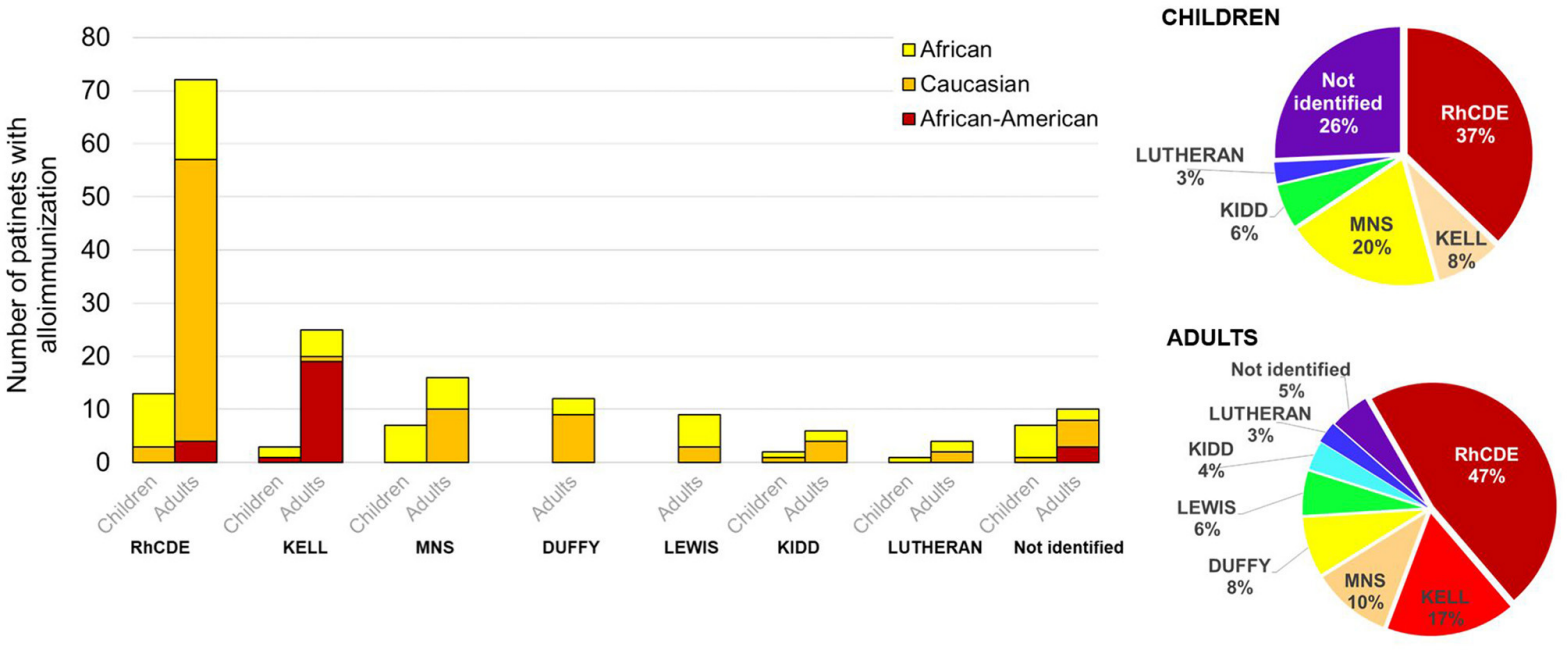
Number of children/adults in SCD patients by antibody system. The pie charts report the overall distribution of the specific antibodies for children and adults. Data are shown as column chart that reports, for each system and differentiating for children/adults, the number of African, Caucasian and African-American patients with the specific antibody.

As shown in Figure 3, alloimmunization predominantly involves RhCDE and Kell systems, with Rhesus (45%) the most represented antibody system, followed by Kell (15%), MNS (12%), Duffy (6.2%), Lewis (4.8%), Kidd (4.3%) and Lutheran (2.7%). In addition, antibodies against minor antigens (0.8%) and antibodies not better identified (8.8%) were also detected. Of interest, anti-e antibodies were found in two e-negative African SCD patients. When we considered alloimmunization profile and patients' age, a higher rate (26 vs. 6%) of alloantibodies with non-identified specificity in children's group was observed (Figure 3, right pie-charts).

Eleven patients (8.1%) with alloimmunization developed a delayed hemolytic transfusion reaction (DHTR) (8 females and 3 males) with a median age of 39 years (IQR: 18.5–49). Six of these patients had SS genotype (4 Africans, 2 Caucasians), and five were Caucasian with Sβ genotype. In 4 out of the 11 patients, a single antibody (D, C, Cw, Jsb) was detected, and in another 4 of the 11 (all females, 3 Caucasians, and 1 African) multiple antibodies (2–4 different specificities) were detected. In 2 patients, antibodies were not clearly identified. Taken together, these data indicate that low alloimmunization events recorded in both African and Caucasian patients might most likely be related to the donor-recipient exact matching for ABO, Rhesus and Kell antigen for RBC compatibility.

## Discussion

Our study highlights the complexity of the clinical management of SCD manifestations throughout the patients' journey. The peculiar multi-ethnic profile of the Italian SCD population, with a roughly equal proportion of Caucasian and African patients, is an added value to the analysis of real-life data. In our series, Caucasian SCD patients were generally older than African patients. This is most likely related to the efficacy of the prenatal program for hemoglobinopathies that started in Italy in the 1980s (29, 30). In contrast, African SCD patients were mainly clustered in pediatric and young adult populations, reflecting the voluntary migration fluxes of the last two decades (22, 23, 31). Indeed, the presence of elderly Caucasian SCD patients represents a unique SCD cohort, providing important information on the combinatory effects of aging and SCD on disease natural history (32, 33).

In our study, the transfusion approach was similar in SS and S/β patients in terms of ATR and CTR regimens, and the percentage of patients with S/β+- and S/β° who were treated with ATR and CTR was similar. Both CTR and ATR were used more frequently in SS patients compared with either S/β+ or S/β°- patients (34). The main reasons for ATR were similar to those described by Rees et al. (35).

Notably, we found that SS African patients were treated with ATR more than Caucasian patients with S/β genotypes. This might be related to (i) patients' low social status; (ii) patients' limited access to family practitioners, generally related to troubles in speaking Italian. All these factors might contribute to the higher access to an emergency department for African SCD patients during acute events when compared to Caucasian SCD subjects (10).

Simple transfusions were the main transfusion strategy used during CTR, and single top-up transfusions were the main one in the acute setting. Manual and automatic exchange transfusions were used more in CTR than in ATR. This might be related to: (i) difficulties in vein access during VOCs; (ii) absence of expertise; (iii) overnight acute events (36, 37).

Of note, we found a subset of patients still symptomatic for acute SCD-related clinical manifestations under treatment with HC, highlighting the biocomplexity of SCD that lies beneath red cell sickling and dehydration (38–44). Indeed, HC partially acts on sickle cell-related inflammatory vasculopathy and chronic inflammation (4, 45–47). Thus, SCD patients considered partial responders to HC require a more intensive treatment, which has until now been represented by the combination of HC and chronic transfusion programs. In our cohort, combination therapy of CTR and HC was administered in a similar percentage of African and Caucasian SS patients. The percentage of SS patients who required long-term combination treatment was higher than the proportion with S/β°-S/β+- genotypes. Considering chronic therapies (CTR and HC), we confirmed that SC patients needed less intensive treatment than other SCD genotypes (14). Thus, our data highlight the presence of a subset of SCD patients with severe clinical manifestations resistant to HC treatment. These might now be eligible for new US Food and Drug Administration and European Medicines Agency therapeutic options such as crizanlizumab, a humanized antibody against P-selectin, or voxelotor, a novel oral anti-sickling agent, alone or in combination with HC (41, 42, 48–52).

In our cohort, alloimmunization was identified in SCD patients aged between 18 and 40 years; those aged <18 years had a lower alloimmunization events than those aged ≥18 years, most likely because they had a shorter transfusion history. A higher proportion of Caucasian patients (10.3%) than African patients (6.6%) had alloimmunization, and among these, Caucasian patients again were older than African patients (median 41 vs. 20 years).

Although the alloimmunization rate was not available due to the study's retrospective design, the low alloimmunization events in both African and Caucasian patients, might reflect the satisfactory level of donor-recipient exact matching for ABO Rhesus and Kell antigen for RBC compatibility, consistent with the Italian guidelines for children and adults released on 2014 (11, 15). Antigen mismatch between donor and recipient is the basis for antibody formation, as the recipient recognizes those antigens as non-self and, thereby, an immune response might be elicited (53, 54). Transfused SCD patients have the highest rates of RBC alloimmunization over their lifetimes (up to 40–50%), compared to fewer than 5% of other transfused individuals with either thalassemia major or myelodysplasia (55, 56). Detection of alloantibodies may make locating compatible RBC units for future transfusion difficult, delayed, and sometimes impossible (54). Potential reasons for alloimmunization include (i) number of donor exposures; (ii) antigenic mismatches between donor RBCs and recipient RBCs related to ethnicity (mainly between Caucasian donors and African descent recipients); (iii) the inflammatory state of recipients (57). SCD is characterized by a chronic inflammatory state, worsening during acute VOCs (57, 58). Thus, SCD patients transfused in their baseline state of health are thought to be less likely to become alloimmunized than patients transfused in a state of inflammation, such as during acute VOCs (58). Studies in SCD patients have shown that having an acute chest syndrome at the time of transfusion is a significant risk factor for becoming alloimmunized (53, 58). In our cohort, the antibodies identified were mainly of RhCDE and Kell systems, as reported in studies of transfused SCD patients from the United States and Brazil (9). Different antibodies systems (i.e., MNS and Kidd) and different frequencies of alloimmunization have been recently described in SCD patients in French Guiana when compared to our cohort or those from the United States or Brazil (18). These might reflect the heterogeneity of donor and recipient populations in different geographical settings, which increases the complexity of data analysis and comparison.

Our study presents some limitations: (i) the retrospective design of the study, which did not allow us to evaluate the alloimmunization rate; (ii) lacking data on patients' transfusion history before their arrival to the comprehensive centers for hemoglobinopathies; (iii) no record on patients' mortality.

In conclusion, our study highlights that transfusion regimens are still crucial as intensive treatments for both acute and chronic SCD-related complications. In our cohort, HC was offered to symptomatic SCD children in agreement with national recommendations (10, 15). This clearly favors a more extensive use of transfusion approaches in the pediatric setting in our cohort in case of acute complications. Since alloimmunizations remains a barrier for safe and effective transfusion of patients with SCD, a careful evaluation of RBC transfusion guidelines transfusion becomes a key factor, along with the implementation of transfusion protocols (such as phenotypically-matched RBCs: RhCDE and Kell), genotyping of patients and donors to identify RBC antigenic variants and maintenance of records on patient transfusion history (59, 60). In addition, dissemination of the knowledge on management of DHTR in patients with SCD, might positively impact patients' outcome (61). These measures are expected to reduce the occurrence of alloimmunization as well as the incidence of delayed hemolytic transfusion reactions, optimizing the management of SCD subjects of different descent throughout the patients' journey.

## Data Availability Statement

The original contributions presented in the study are included in the article/Supplementary Material, further inquiries can be directed to the corresponding author.

## Ethics Statement

The studies involving human participants were reviewed and approved by Ethics Committee of Fondazione IRCCS Ca' Granda, Ospedale Maggiore Policlinico of Milan, Italy. Written informed consent to participate in this study was provided by the participants' legal guardian/next of kin.

## Author Contributions

GG, LDF, and GLF contributed to the conceptualization and design of the study, acquisition and curation of the data, contributed to data analysis and interpretation, writing, critical appraisal and comments, and reviewing and editing. LS, DV, NM, PB, AV, MC, GL, and VV contributed to the conceptualization and design of the study, acquisition and curation of the data, and critical appraisal and comments. PR, VP, AQ, LN, GR, MA, RR, DD'A, EF, SMar, FA, FB, ER, AS, SC, GC, FG, RL, PG, GB, AF, SMac, PM, MM, RO, FL, MB, RC, RD, AP, PC, CF, GP, and LB contributed to acquisition and curation of the data. BG and FP contributed to data analysis and interpretation. All authors have read and agreed to the published version of the manuscript.

## Funding

Financial support for Medical Editorial Assistance was provided by Novartis Farma SpA. Novartis Farma SpA was not involved in the study design, collection, analysis, interpretation of data, and the writing of this article or the decision to submit it for publication.

## Conflict of Interest

The authors declare that the research was conducted in the absence of any commercial or financial relationships that could be construed as a potential conflict of interest.

## Publisher's Note

All claims expressed in this article are solely those of the authors and do not necessarily represent those of their affiliated organizations, or those of the publisher, the editors and the reviewers. Any product that may be evaluated in this article, or claim that may be made by its manufacturer, is not guaranteed or endorsed by the publisher.

## Supplementary Material

Supplementary Figure S1 Flow-chart of study population. Pts, patients; SCD, sickle cell disease.

Supplementary Figure S2 Distribution of the indications for transfusion therapy regimen for children and adults, **(A)** Indications for acute transfusion regimen (ATR). **(B)** Indications for chronic transfusion regimen (CTR). Data are shown as counts of the indications for ATR and CTR. ACS, acute chest syndrome; ATR, acute transfusion; CTR, chronic transfusion; HC, hydroxycarbamide; MOFs, multi-organ failures; VOCs, vaso-occlusive events.

Supplementary Figure S3 **(A)** Distribution of new antibodies developed in after the arrival at the center for sickle cell disease (SCD) patients with pre-existing (up) and without (low) pre-existing antibodies. **(B)** Number of SCD children/adults patients with alloimmunization by ethnicity. **(C)** Number of SCD children/adults patients with alloimmunization presenting single, multiple or unidentified antibody/ies. Data are shown as pie and column charts with the number of patients with alloimmunization.
