# Supplementary figures and images for: Transfusional Approach in Multi-Ethnic Sickle Cell Patients: Real-World Practice Data From a Multicenter Survey in Italy
Source: Front Med (Lausanne). 2022 Mar 16;9:832154. doi: 10.3389/fmed.2022.832154 (PMC8967327; doi:10.3389/fmed.2022.832154)

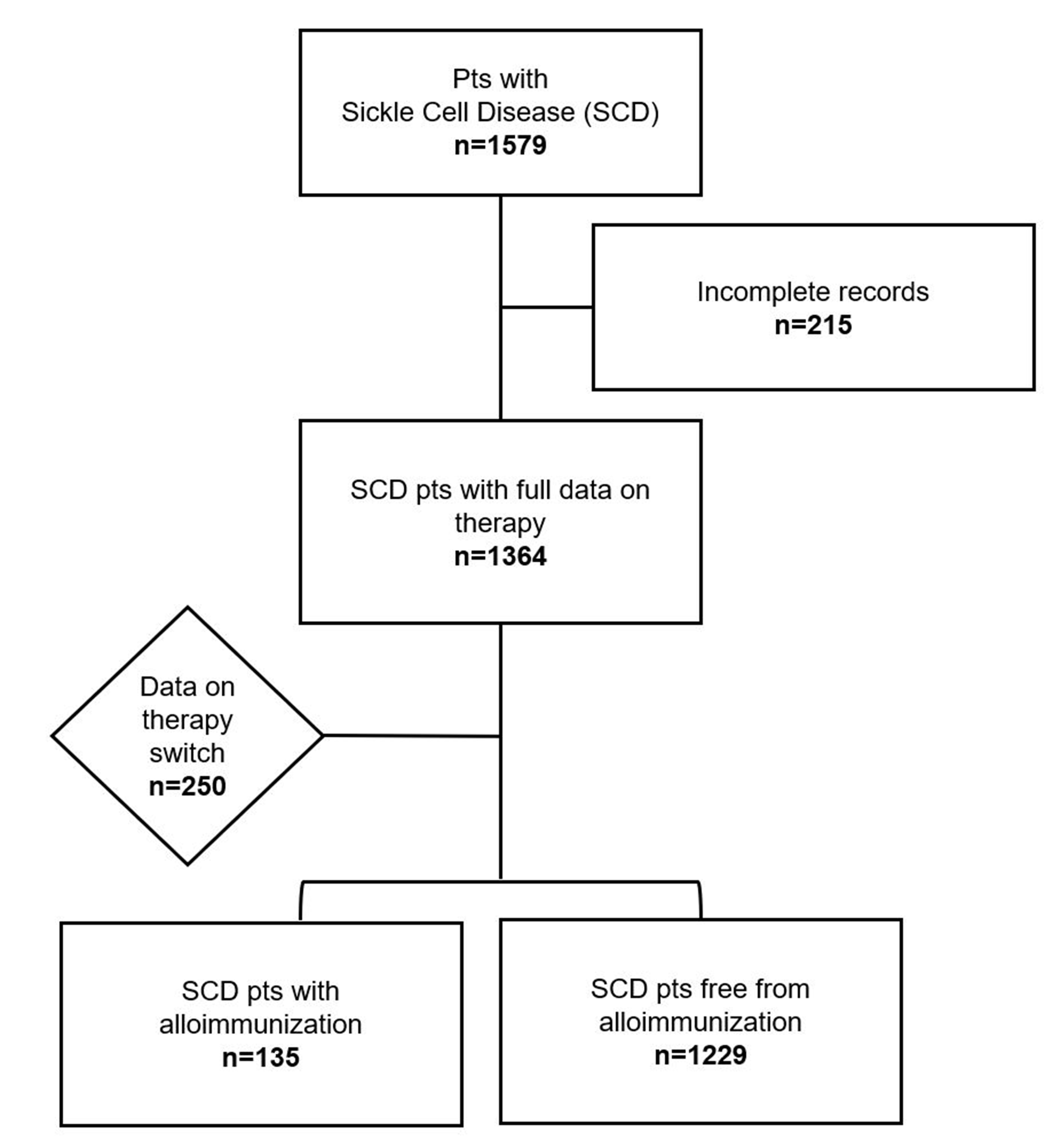

Supplement: Supplementary Figure S1 — Flow-chart of study population. Pts, patients; SCD, sickle cell disease. [file Image_1.JPG]

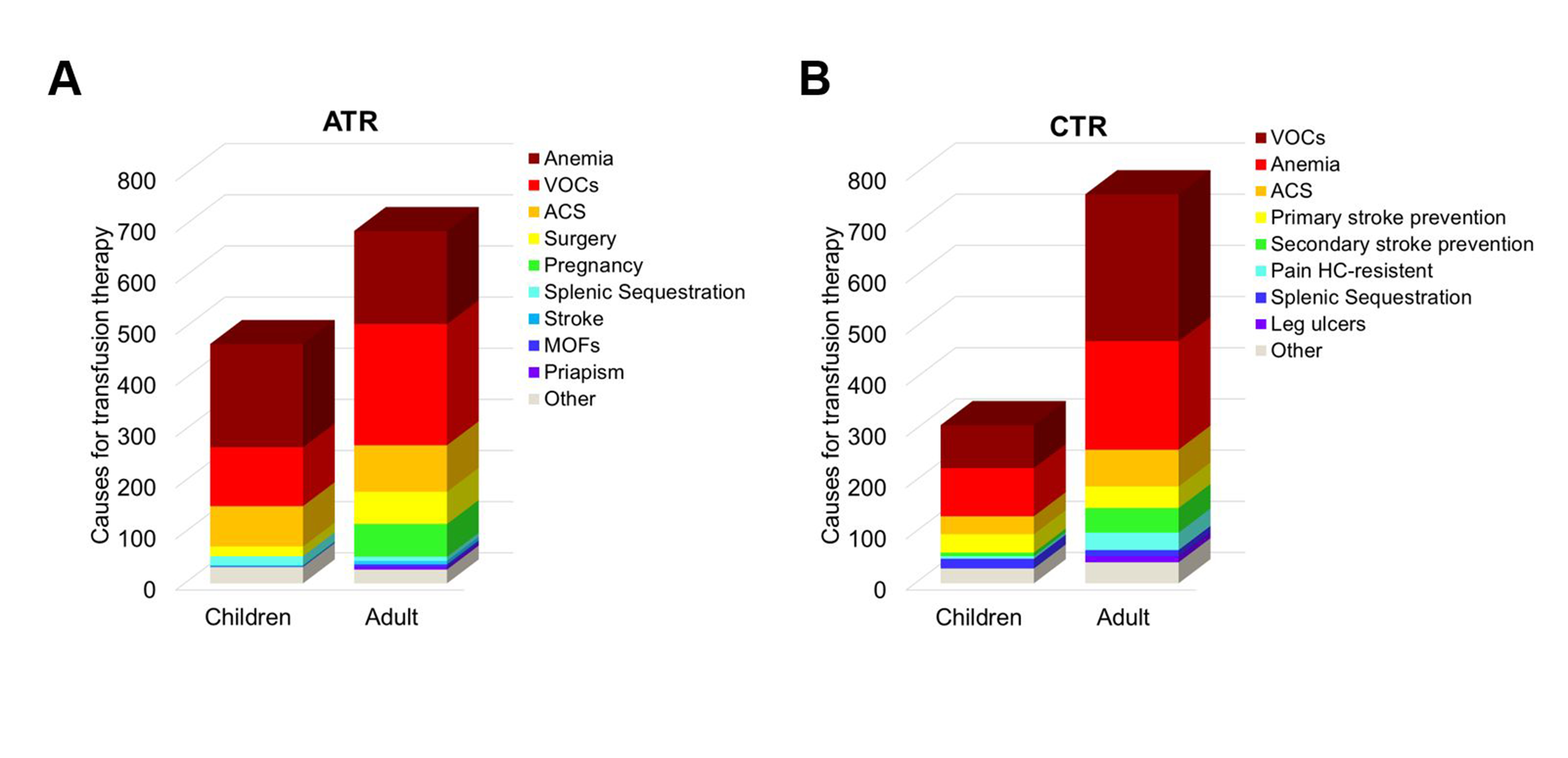

Supplement: Supplementary Figure S2 — Distribution of the indications for transfusion therapy regimen for children and adults, (A) Indications for acute transfusion regimen (ATR). (B) Indications for chronic transfusion regimen (CTR). Data are shown as counts of the indications for ATR and CTR. ACS, acute chest syndrome; ATR, acute transfusion; CTR, chronic transfusion; HC, hydroxycarbamide; MOFs, multi-organ failures; VOCs, vaso-occlusive events. [file Image_2.JPG]

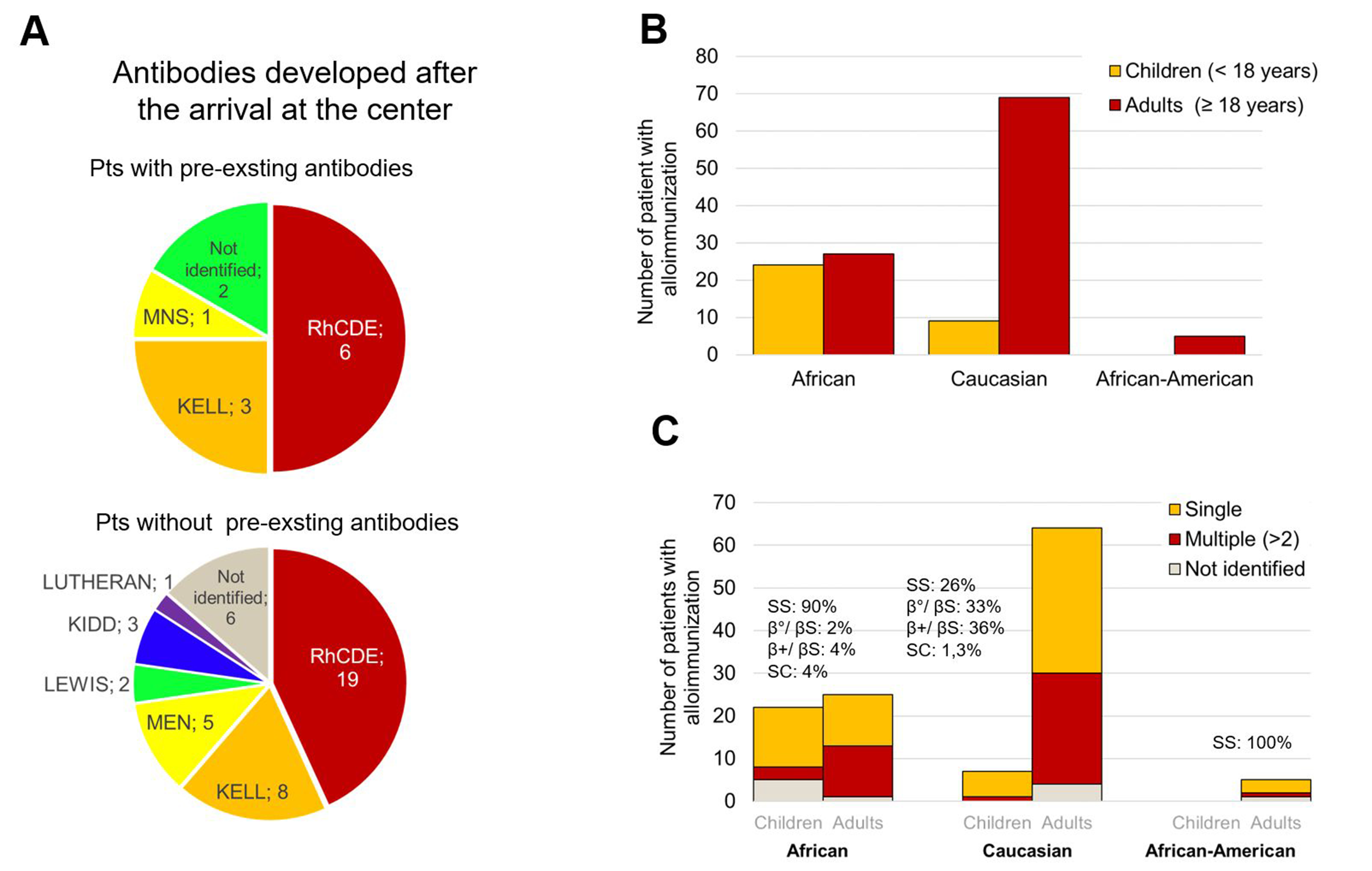

Supplement: Supplementary Figure S3 — (A) Distribution of new antibodies developed in after the arrival at the center for sickle cell disease (SCD) patients with pre-existing (up) and without (low) pre-existing antibodies. (B) Number of SCD children/adults patients with alloimmunization by ethnicity. (C) Number of SCD children/adults patients with alloimmunization presenting single, multiple or unidentified antibody/ies. Data are shown as pie and column charts with the number of patients with alloimmunization. [file Image_3.JPG]
